# Targeting and activation of macrophages in leishmaniasis. A focus on iron oxide nanoparticles

**DOI:** 10.3389/fimmu.2024.1437430

**Published:** 2024-08-15

**Authors:** Carmen Palomino-Cano, Esther Moreno, Juan M. Irache, Socorro Espuelas

**Affiliations:** ^1^ Department of Pharmaceutical Sciences, School of Pharmacy and Nutrition, University of Navarra, Pamplona, Spain; ^2^ Navarra Medical Research Institute (IdiSNA), Pamplona, Spain

**Keywords:** leishmania, iron oxide nanoparticles, macrophages, host-directed therapies, target, targeted delivery

## Abstract

Macrophages play a pivotal role as host cells for *Leishmania* parasites, displaying a notable functional adaptability ranging from the proinflammatory, leishmanicidal M1 phenotype to the anti-inflammatory, parasite-permissive M2 phenotype. While macrophages can potentially eradicate amastigotes through appropriate activation, *Leishmania* employs diverse strategies to thwart this activation and redirect macrophages toward an M2 phenotype, facilitating its survival and replication. Additionally, a competition for iron between the two entities exits, as iron is vital for both and is also implicated in macrophage defensive oxidative mechanisms and modulation of their phenotype. This review explores the intricate interplay between macrophages, *Leishmania*, and iron. We focus the attention on the potential of iron oxide nanoparticles (IONPs) as a sort of immunotherapy to treat some leishmaniasis forms by reprogramming *Leishmania*-permissive M2 macrophages into antimicrobial M1 macrophages. Through the specific targeting of iron in macrophages, the use of IONPs emerges as a promising strategy to finely tune the parasite-host interaction, endowing macrophages with an augmented antimicrobial arsenal capable of efficiently eliminating these intrusive microbes.

## 1 Introduction

Flagellate protists of the genus *Leishmania* are the etiological agents of leishmaniasis, a vector-borne parasitic disease associated with a pronounced immune system dysfunction. The World Health Organization (WHO) classifies this condition as one of the twenty neglected tropical diseases (NTDs) that threaten more than 1.7 billion people and disproportionately affect the poorest strata of the population (1).

The term leishmaniasis encompasses a broad spectrum of clinical symptoms and pathologies, ranging from asymptomatic infections and self-healing skin lesions (cutaneous leishmaniasis, CL) to more severe forms such as mucocutaneous leishmaniasis (MCL) or visceral leishmaniasis (VL), which can be life-threatening if left untreated. This heterogeneity of clinical manifestations is the result of a complex interplay between the immune response and genetic characteristics of the host, and parasite characteristics such as infectivity and virulence (2).

The burden of this disease is immense, being endemic in 99 countries in tropical and subtropical areas worldwide. According to the WHO, there were approximately 250,000 new cases of leishmaniasis worldwide in 2022 although the actual burden of the disease is often underestimated due to under-reporting in many of the affected areas (3, 4). At the global level, there is a general increasing trend in the number of new cases, associated with environmental changes such as deforestation, the building of dams, irrigation schemes, and urbanization (4).

The unavailability of a human vaccine and effective vector control programs render chemotherapy the only option to control leishmaniasis. However, available treatments are expensive, impractical, toxic, and, subject to drug resistance that lessens their efficacy (1, 3). Importantly, drug resistance is the main cause of leishmanicidal chemotherapy failures, as the amazing genetic plasticity of the parasite allows it to adapt easily to new and challenging environments, such as drug pressure (5). Consequently, there is an urgent need for alternative therapeutic strategies that address these limitations.

Once in the host, and although *Leishmania* can infect different cells, macrophages are their preferred and final target, being indispensable for its survival, replication, and differentiation. In their interior, the extracellular forms of the parasite -promastigotes- differentiate into the intracellular ones -amastigotes. They divide by binary fission until reaching a high number, leading to the cellular lysis and its liberation into the blood flow. This release finally cause the infection of surrounding macrophages and thus expanding the infection (6).

Macrophages are phagocytic cells with enormous phenotypic plasticity that allows them to carry out a plethora of different functions, ranging from tissue repair to eliminating invasive microorganisms. A wide spectrum of distinct phenotypes exists, typically represented by the pro-inflammatory M1 macrophage and the anti-inflammatory M2 macrophage, that are determined by the presence of signals (e.g., cytokines) in the local microenvironment of the cell. This functional dichotomy includes the expression of specific markers, a particular cytokine pattern, and different types of energy metabolism (7).

Although the interior of a macrophage constitutes a hostile environment for any microbe or parasite, *Leishmania* adeptly manipulates host cell signaling, metabolism, and immune functions steering the macrophage toward an anti-inflammatory phenotype similar to M2 (8). This intricate interplay between macrophages and *Leishmania* unveils a dynamic relationship critical for determining the course of infection. The balance between M1/M2 phenotypes emerges as a pivotal factor in infection dynamics, as illustrated by studies in mouse models. C57BL/6 mice infected with *L. major* mice are resistant to infection, triggering a robust Th1 response, which is aligned with the pro-inflammatory M1 phenotype (interferon-gamma (IFN-γ) and interleukin-12 (IL-12) production). In contrast, the vulnerability of BALB/c mice is marked by a Th2 response, paralleling the anti-inflammatory M2 phenotype (9–11). This correlation underlines the importance of macrophage polarization in establishing a potent immune response capable of controlling *Leishmania* infection. Thus, addressing macrophage polarization either directly or indirectly emerges as a promising strategy for combating leishmaniasis, which is classified within host-directed therapies (HDT). These therapies utilize agents that are not microbicides per se, but rather their action is directed by modulating host cell immunity (12). This approach has the enormous advantage of being refractory to drug resistance that threatens the efficacy of current leishmanicidal therapies. Leveraging the sensitivity of *Leishmania* to endogenous microbicidal mechanisms of macrophages, various agents have been used to unlock the microbicidal functions that the parasite is able to encrypt, including toll-like receptors (TLR) agonist like Imiquimod and CpGs oligonucleotides (13–15).

One of the most intriguing battles between *Leishmania* and the macrophage is the competition for iron, an essential element for the survival and functions of both. On the one hand, *Leishmania* has developed sophisticated mechanisms to acquire the iron necessary for its proliferation. On the other hand, it must be very careful in its eagerness to increase macrophage iron levels due to the close relationship between cellular iron metabolism and antimicrobial mechanisms, such as the generation of reactive oxygen species (ROS) by the Fenton reaction (16).

With this review, we aim to provide insight into the intricate tug-of-war between *Leishmania* and the macrophage, with a particular emphasis on the battle for iron, recapitulating the works that use its modulation as a way to enhance the microbicidal response of the macrophage. In addition, we will focus on iron oxide nanoparticles (IONPs), already approved by the Food and Drug Administration (FDA) for other applications (17), as a host-targeted therapy against leishmaniasis. Our interest in IONPs is based on their ability to address three pivotal aspects crucial for treating a neglected disease like leishmaniasis: i) they influence the immunology of macrophages rather than directly targeting the parasite, which mitigates the risk of drug resistance development; ii) as nanoparticles (NPs), they offer the prospect of decreased toxicity and improved efficacy by selectively accumulating in macrophages, which mimics the final fate of the parasites; iii) their repurposing could significantly enhance patient accessibility by substantially lowering development and approval costs.

## 2 The *Leishmania*-macrophage tug-of-war. A tale of persuasion, manipulation, and exploitation

### 2.1 The macrophage: a lethal chamber for *Leishmania*

Once *Leishmania* is ingested by macrophages, it docks into the parasitophorous vacuole, which fuses with lysosomes, transforming into a phagolysosome with microbicidal properties. It is characterized by an acid pH (induced by vacuolar ATPase, which pumps protons into it) and many proteases (such as cathepsins) responsible for its proteolytic degradation (16, 18).

In addition to sequestering the parasite in an inhospitable terrain such as the interior of a phagolysosome, macrophages produce a variety of toxic compounds that help to destroy the phagocytosed microorganism: nitric oxide (NO), and ROS. Within the phagosome membrane, the orchestrated activity of the complex NADPH oxidase (Nox2) starts the respiratory burst, a pivotal phase in the macrophage’s offensive. Nox2 initiates the production of the superoxide ion from molecular oxygen, an elemental maneuver in the macrophage’s armamentarium against intruding pathogens. This superoxide ion is converted into hydrogen peroxide (H_2_​O_2_) through the catalytic prowess of the enzyme superoxide dismutase (SOD). Simultaneously, additional reactions unfold, giving rise to toxic substances like hypochlorous acid (HOCl), further amplifying the macrophage’s arsenal in the ongoing cellular warfare (19).

Concomitantly, the macrophage orchestrates the induction of inducible nitric oxide synthase (iNOS). This catalytic maestro transforms L-arginine into L-citrulline with the generation of NO as a by-product. This enzymatic feat unfolds in the presence of immunomodulatory signals such as IFN-γ and tumor necrosis factor-alpha (TNF- α) (20). The dynamic interplay between NO and ROS ushers forth the generation of derivatives like peroxynitrite (OONO). These formidable agents of cellular damage covalently bind to DNA, inducing deamination of nucleotide bases and precipitating diverse alterations, that ultimately execute a lethal blow to the pathogens (21). Additionally, the liberation of transition metals such as iron from proteins in the phagosome can result in Fenton chemistry. It is a catalyzed oxidation reaction involving iron, resulting in the production of hydroxyl radicals in the presence of hydrogen peroxide (16).


$$\text{F}{\text{e}}^{2+}+{\text{H}}_{2}{\text{O}}_{2}->\text{F}{\text{e}}^{3+}+\text{O}{\text{H}}^{-}+\cdot \text{OH}$$

This oxidative burst and NO generated by macrophages are key to restricting the intracellular growth of *Leishmania*. The inhibition of iNOS hampers this effect, which emphasize the indispensability of NO production in control infection (20, 22, 23). Clinical observations revealed an inverse correlation between iNOS expression and the severity of *L. tropica* infections (24). Additionally, studies inhibiting ROS production in *L. braziliensis*-infected monocytes underscored the crucial role of ROS, as their inhibition increases parasite survival (25). Collectively, NO and ROS stand as the main microbicide molecules orchestrating macrophage defenses, pivotal for effective *Leishmania* control.

In parallel with these microbicidal molecules, macrophages also secrete mediators to engage other types of immune cells and enhance the overall offense. Macrophages release proinflammatory cytokines such as TNF-α, IL-12, and IFN-γ, acting as alarm signals (26). These cytokines not only amplify the microbicidal activity of the macrophages themselves but also recruit additional immune cells, such as lymphocytes to participate in the defense, promoting a Th1 response. On the other end of the spectrum are IL-4 and IL-13, which are associated with a Th2 response and, therefore, with susceptibility to infection by not activating macrophages properly (27).

If none of these strategies succeed in controlling the infection, macrophages resort to an extreme measure: they induce their own sacrifice to prevent parasites’ spread and preserve the organism’s integrity. During the apoptotic process, signaling cascades are activated, leading to a controlled death, and avoiding the uncontrolled release of parasites into the surrounding environment (28, 29). This act of self-destruction represents a remarkable example of the immune system’s adaptive strategies in the face of persistent infections.

### 2.2 Survival strategies of *Leishmania* inside macrophages

*Leishmania* has evolved sophisticated ways to resist the array of microbicidal mechanisms generated by the macrophage, namely the oxidative stress, the recruitment of other immune cells and the process of apoptosis.

#### 2.2.1 Quenching the flames: *Leishmania*´s control of oxidative stress

**Figure 1:**
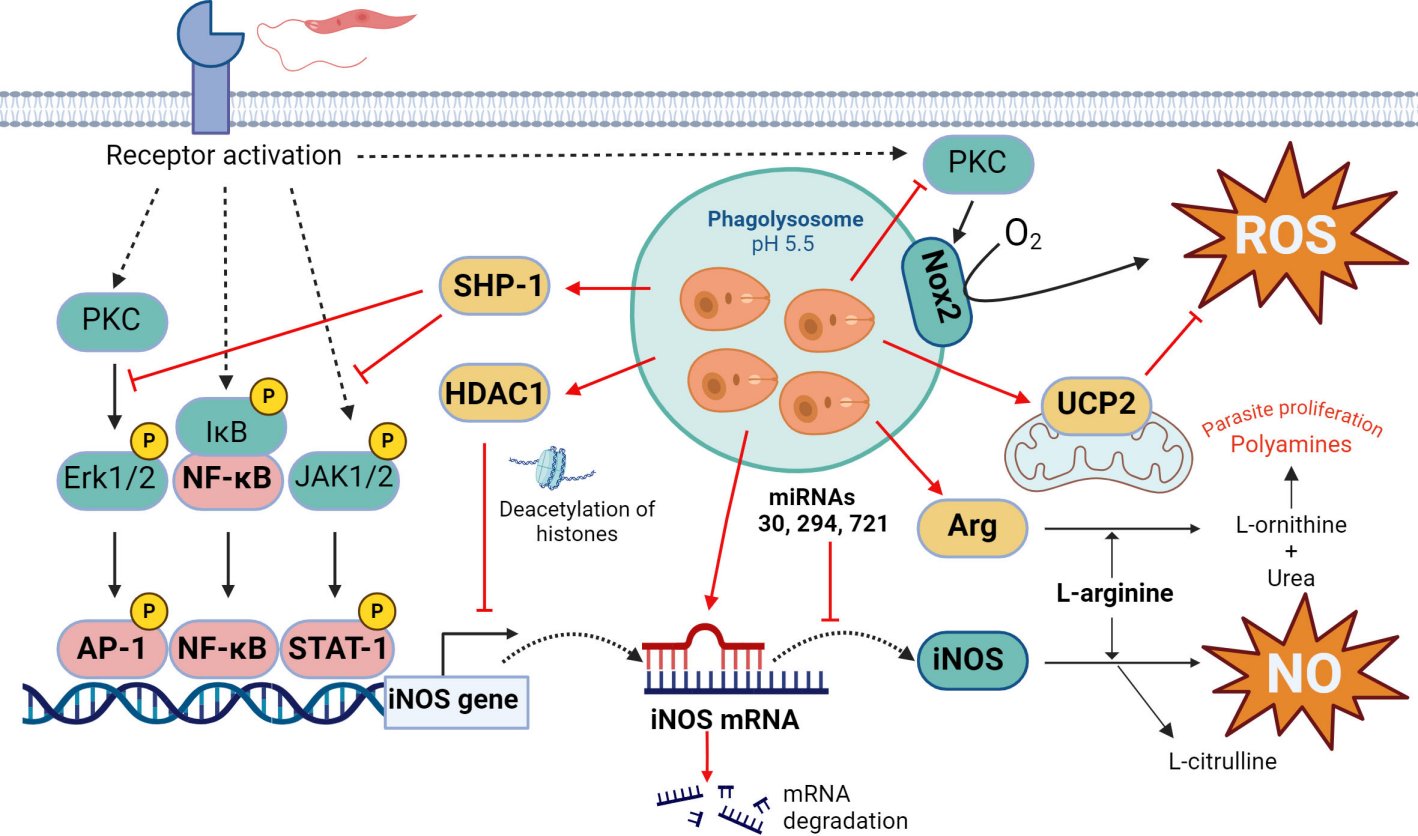
Oxidative response in *Leishmania* infection. The key leishmanicidal molecules produced by the macrophage are ROS and NO, although *Leishmania* parasite attempts to stop their generation at different levels. On the one hand, *Leishmania* can inhibit iNOS expression through multiple mechanisms, including JAK2-STAT1 and ERK1/2-AP1 signaling pathways interference, chromatin condensation through HDAC1 recruitment, negative regulation by microRNAs, and substrate competition through overexpression of the enzyme arginase. In the case of ROS, the parasite prevents Nox2 complex formation and PKC activation and induces mitochondrial UCP2. Actions that *Leishmania* triggers to counteract the production of ROS/NO by the host cell are indicated in red. Figure created with biorender.com.

The oxidative burst is crucial to control *Leishmania* proliferation by the macrophage, so the main goal of the parasite is to protect itself from damage caused by these oxidative molecules (ROS and NO), which can eliminate it. Stopping this macrophage offensive is critical for this growth, as it represents a paradigmatic case of how the parasite exerts its manipulation on the host at all levels of the genetic decoding process (genome -> transcriptome -> proteome), underlining its significant importance (**Figure 1**).

First, *Leishmania* prevents transcription of the iNOS gene by interfering with the signaling pathway that leads to the binding of the transcription factor STAT-1 to its promoter. Thus, *Leishmania* inhibits Janus kinase 2-signal transducer and activator of transcription 2 (JAK2-STAT1) and extracellular-regulated kinase 1-activator protein 1 (ERK1/2-AP1) signaling cascades, thereby preventing iNOS expression (30, 31). Moreover, it activates the Src homology 2-containing phosphatase-1 (SHP-1) (32), which prevents phosphorylation of both JAK2 and ERK1/2 and downstream phosphorylation of the transcription factors STAT1 and AP1, which control enzyme expression (30, 33). Derivate of knockout mice for SHP1 showed a higher generation of NO and consequently, a higher efficiency in the control of the infection by *L. donovani* (34) (**Figure 1**).

Furthermore, even if these signaling pathways are activated, *Leishmania* can block access of the transcription machinery to the iNOS gene promoter. It induces chromatin condensation in that genomic region by modifying the expression and activity of histone deacetylase 1 (HDAC1) which methylates lysine 9 of histone 3 (35). The precise mechanism by which the parasite accomplishes this effect remains not fully understood, although competition for the activity of histone-modifying enzymes between histones released by the parasite could occurs. Additionally, these histones themselves are capable of independently altering chromatin structure (36, 37). A similar effect has been described for *L. amazonensis*, which, by activating the phosphatidylinositol 3-kinase/protein kinase B (PI3K/Akt) pathway, activates the p50/p50 transcriptional repressor of the nuclear factor kappa-light-chain-enhancer of activated B cells (NF-κB) family, which binds to the iNOS promoter and prevents its transcription (38).

The expression of iNOS is also controlled by microRNA (miRNAs), which are small non-codifying RNAs that interact with regions 3’ of the messengers. This leads to its degradation and, thus, prevent their translation. *Leishmania* infection has been shown to interfere with the regulation of miR-30, miR-294 and miR-721, which bind the iNOS messenger and reduce cellular levels of the enzyme (39, 40) (**Figure 1**).

Even if iNOS are produced, the parasite has another way to decrease NO production. *Leishmania* manages to upregulate arginase, an enzyme that competes for the same substrate as the iNOS, L-arginine. Wilkins-Rodríguez et al. attribute an increase in the virulence of *L. mexicana* strains in which arginase enzyme activity is higher compared to iNOS (41). In addition, arginase hydrolyzes L-arginine to generate urea and L-ornithine, a precursor in the biosynthesis of polyamines which is necessary for *Leishmania* proliferation (42), modifying the macrophage metabolite pool in its favor (**Figure 1**).

Regarding ROS, *Leishmania* also controls their production at different levels*. Leishmania* prevents the assembly of Nox2 complex in the vacuole membrane, inhibiting the generation of ROS and favoring its survival (43). This protection is mediated by the metalloprotease gp63 present in their membrane, which directly cleaved the vesicle-associated membrane protein 8 (VAMP8), responsible for the recruitment of gp91^phox^- component of Nox2- to the phagolysosome (44). At the same time, through the lipophosphoglycan (LPG) and glycoprotein 63 (gp63), *L. major* interrupts the activation of protein kinase C (PKC) (42, 45), a kinase that stimulates the activity of Nox2 (46). Moreover, *L. donovani* induces the expression of the uncoupling protein 2 (UCP2), a protein of the mitochondria membrane that acts as a negative regulator of the production of ROS (47). In fact, silencing UCP2 by small interfering RNA (siRNA) increases ROS production and leads to reduced parasite survival (48) (**Figure 1**).

Apart from these ways in which *Leishmania* minimizes the production of ROS and NO, it also manages to enhance the host antioxidant response by upregulating the transcription factor NF-E2-related factor 2 (NRF2) (49, 50). This transcriptional factor (TF) binds to genes promoters of different antioxidant enzymes such as thioredoxin (TXN) or some glutathione S-transferases (GSTs) (51).

#### 2.2.2 Interfering communications: *Leishmania*´s sabotage of antigen presentation and cytokine production

**Figure 2:**
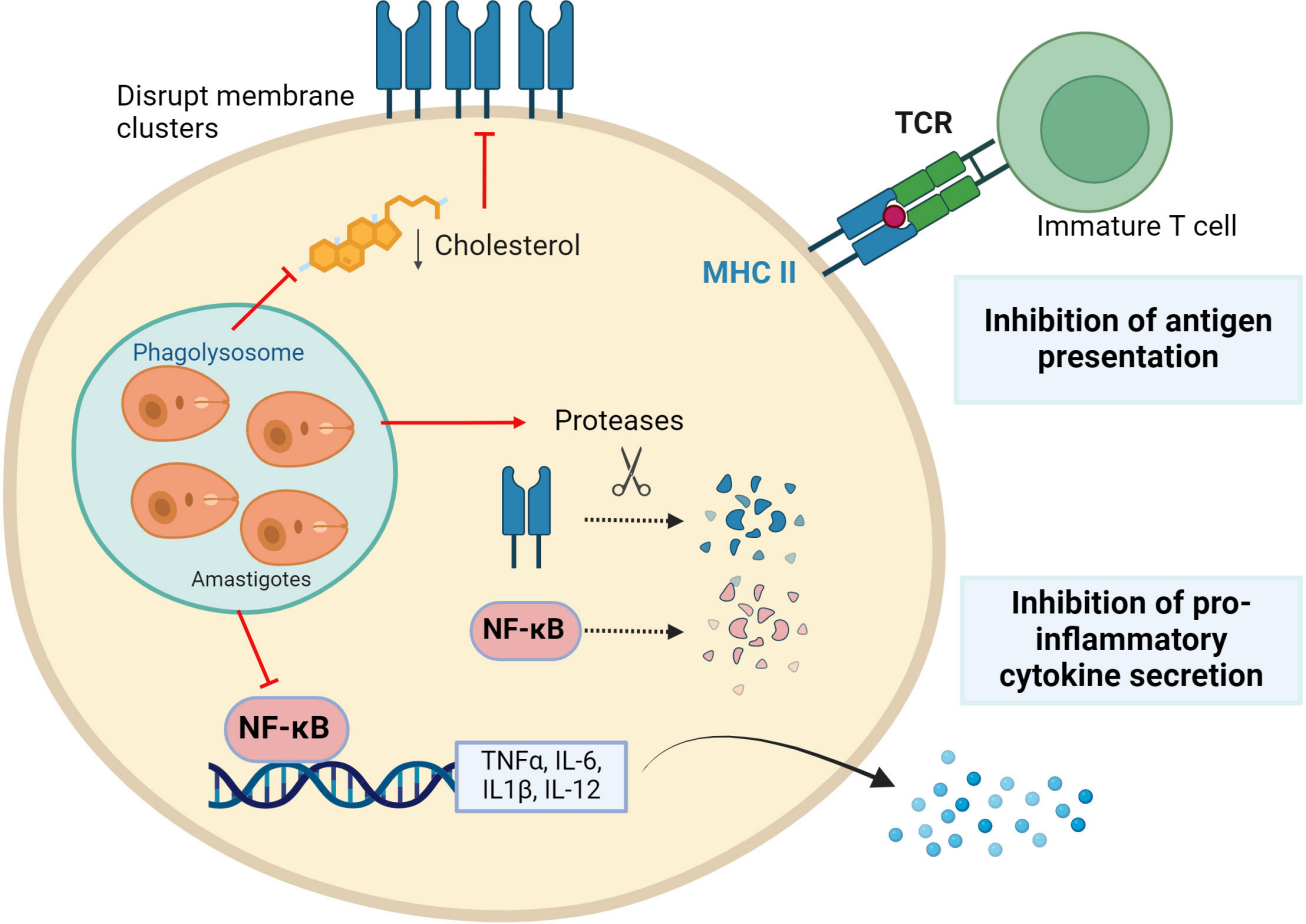
*Leishmania* parasites evading immune surveillance. *Leishmania* reduces antigenic presentation by preventing Major Histocompatibility Complex-II (MHCII) clusters formation by modifying membrane fluidity and inducing their protease-mediated degradation. In addition, the parasite induces proteases to degrade the transcription factor NF-kB, which is essential for the expression of pro-inflammatory cytokines. Figure created with biorender.com.

The collaboration between cells of the immune system is essential to mount a potent response to an invader. Thus, in addition to protecting itself from the microbicidal molecules of macrophages, *Leishmania* also prevents other cell types from entering the fray by interfering with antigen presentation and cytokine production - mediators responsible for intercellular communication (**Figure 2**) (52).

Antigens are presented less efficiently in *Leishmania*-infected macrophages than in uninfected macrophages (53), enabling evasion from T cell detection. This is accomplished by lowering the presence of major histocompatibility complex (MHC) molecules, especially MHC-II, on macrophage surfaces. In *L. donovani*-infected macrophages, expression of the MHC-II gene is blocked (54, 55). Furthermore, MHC-II protein is broken down by macrophage cysteine proteases, while some are internalized and degraded by parasitic cysteine proteases within amastigotes (56). Additionally, parasites disrupt the antigen processing and MHC binding pathway, which commence in the endoplasmic reticulum (57–59). The MHC II molecules are concentrated in membranes of antigen-presenting cells within lipid rafts, ensuring efficient T-cell activation (60). It has been described that *Leishmania* promotes a decrease in cholesterol in the macrophage membrane, increasing its fluidity and affecting antigen presentation. Thus, even if there are enough peptide-MHC complexes within the cell, they are unable to stimulate T cells due to their inability to form clusters in the lipid raft. Indeed, supplementation of *Leishmania*-infected macrophages with cholesterol restores membrane fluidity and therefore antigen presentation function (61–63).

*Leishmania* also modulates cytokine expression to promote an extracellular pro-parasite environment. The pattern of cytokines secreted by macrophages is very different depending on the infecting species, exhibiting sometimes dual functions. However, in general, all species of *Leishmania* tend to promote an anti-inflammatory phenotype, avoiding detection by surrounding immune cells (52, 64, 65). This is partly because the parasite alters the function of NF-κB, a master regulator that controls the transcription of pro-inflammatory cytokine genes (IL-12, IL-6, IL1-β, and TNF-α). Changes in methylation/acetylation of histones in promoters related to the NF-κB signaling pathway have been described. Concretely an increase in transcripts of pathway inhibitors such as TNF-α induced protein 3 (TNFAIP3) and a decrease in activators like myeloid differentiation primary response 88 (MYD88) and p65 (RelA) have been found. This is correlated with a diminished inflammatory response (37). Additionally, it has been observed that *Leishmania* proteolytically breaks down NF-kB through cysteine peptidase (CPB), affecting the transcription of the IL-12 gene, among others (66, 67).

#### 2.2.3 Dancing with the death: the anti-apoptotic maneuvers of *Leishmania*

Another hurdle for *Leishmania* is to protect its niche because when macrophages, designed to eradicate pathogens, fail in its purpose, they sacrifice themselves by triggering apoptosis, a controlled form of cellular self-digestion. Thus, the parasite employs cunning strategies to inhibit the host’s self-destructive mechanisms.

A key feature of the apoptotic process is DNA fragmentation, often induced by antimicrobial oxidative stress (ROS are apoptosis inducers). In macrophages infected with *Leishmania*, both transcriptomic and proteomic analyses have revealed an increase in DNA repair enzymes (68). This DNA damage activates the p53 protein, recruiting Bax or Bad to the outer mitochondrial membrane, creating a pore and releasing cytochrome C (Cyt C) into the cytoplasm. Cyt C activates the apoptosome protein complex, which initiates the caspase cascade (3, 6, 9) and triggers the final stages of the death process (69).

**Figure 3:**
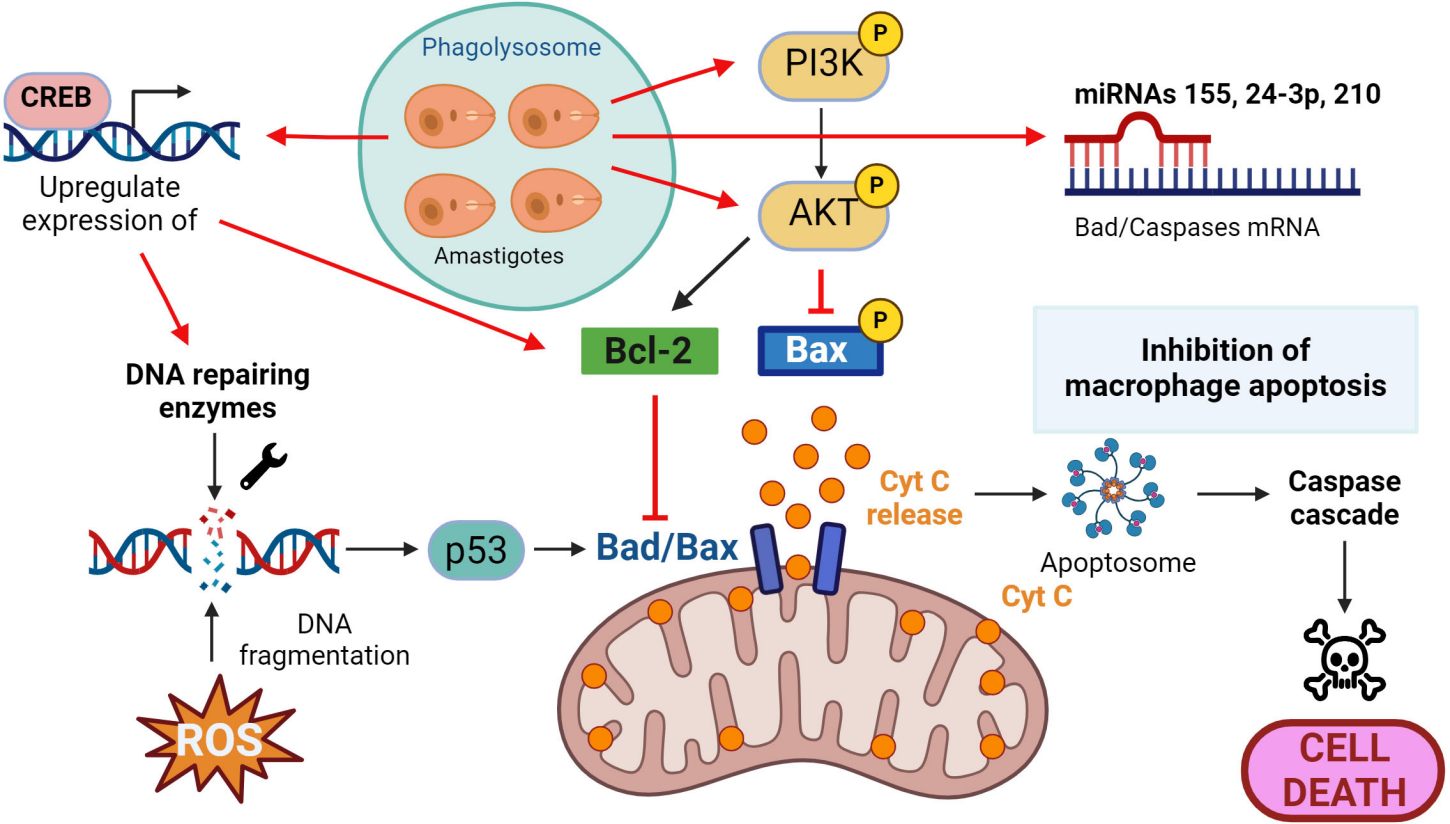
*Leishmania* and macrophage apoptosis. Macrophages activate the self-destruct mechanism in response to DNA fragmentation because of the infective process, and the parasite then implements strategies to inhibit this process. These include upregulation of DNA repair enzymes, activation of the PI3K/Akt pathway to prevent the release of cytochrome C (Cyt C) and promotion of anti-apoptotic proteins such as Bcl-2 and the inhibition of pro-apoptotic proteins such as Bad/Bax. Actions that *Leishmania* triggers to counteract the apoptotic process are presented in red. Figure created with biorender.com.

A primary anti-apoptotic mechanism described in various *Leishmania* species involves the activation of the PI3K/Akt pathway (**Figure 3**). The parasite activates both Akt and PI3K, leading to the phosphorylation and inhibition of Bad, preventing the release of Cyt C into the cytosol (70–72). Furthermore, decreasing Akt levels through the application of siRNAs provoked incapacity of resisting apoptosis in infected macrophages (71).

Like in the other “battlefronts” we’ve explored, *Leishmania* not only hinders the apoptotic process at the signaling cascade level (activating/deactivating phosphorylation processes, for instance) but also deploys its arsenal by interfering at transcriptional and post-transcriptional levels. The parasite promotes the expression of the anti-apoptotic protein B-cell lymphoma 2 (Bcl-2), directly antagonizing the effects of Bad/Bax on the mitochondria (73). Additionally, *Leishmania* acts on transcription factors that regulate the expression of pro-apoptotic genes, such as the cAMP response element-binding protein (CREB), controlling the expression of myeloid cell leukemia 1 (MCL-1), whose effect is similar to the aforementioned Bcl-2 (74). Finally, various studies have also shown that *Leishmania* upregulates miRNAs such as miR-24-3-p, miR-155, or miR-210, which bind to transcripts of caspases 3 and 7 or Bad, inhibiting the apoptotic process (75–77).

## 3 Macrophages and *Leishmania*: a quest for iron, the precious metal

Iron is a transition metal that exist in multiple oxidation states ranging from -2 to +7. This element is essential for living organisms as it serves as a cofactor for a multitude of proteins involved in numerous biological functions such as oxygen transport (hemoglobin), storage and use of oxygen in muscles (myoglobin), DNA synthesis (ribonucleotide reductase) or cellular respiration and electron transport (cytochromes), among others (78).

The key to its biological utility is the interconversion between its most common and biologically relevant species: the divalent ferrous iron (Fe^2+^) and the trivalent ferric ion (Fe^3+^), which allows it to act as a redox catalyst by easily accepting or donating electrons. However, this reactive property also makes it a dangerous element, as it participates in injurious ROS generation by forming part of the catalytic center of enzymes such as xanthine oxidase, Nox2, or lipoxygenases, or directly by the aforementioned Fenton reaction (79).

### 3.1 The importance of iron in macrophage biology: Its role in the polarization game and vice versa

Given the toxic potential of iron accumulation, iron levels must be finely regulated in the body and macrophages are the main cell type regulating iron homeostasis. Interestingly, in addition to their role in iron efflux at systemic level, macrophages have recently been described as “ferrostats”, capable of sensing and regulating iron availability at local level, assisting in tissue and cellular function (80).

At systemic level, macrophages play a crucial role in iron recycling, supporting the synthesis of hemoglobin required for the daily production of approximately 200 billion of red blood cells. Hemophagocytic macrophages are responsible for removing senescent red blood cells, facilitating iron reuse [detailed in (81, 82)]. Intracellularly, macrophages internalize iron through various receptors such as the transferrin receptor (TfR) and the hemoglobin-haptoglobin (CD163), and they also have transporters such as ferroportin (FPN) that export iron to the plasma. In addition, macrophages can either store iron, mostly complexed with ferritin, or released a free fraction in the cytoplasm, known as the labile iron pool (LIP). This free iron fraction, whose concentrations vary from nanomolar to millimolar, is metabolically accessible and is the key to macrophage metabolism and function (82, 83). Most of the intracellular iron is used by macrophages for its incorporation into iron-binding proteins, contributing to diverse effector functions such as mitochondrial respiration, DNA repair, or immune responses defense against pathogens (including the abovementioned Nox2 and iNOS, for example) (84–86).

In addition, iron is involved in the post-transcriptional regulation of a multitude of genes via the iron regulatory proteins/iron-responsive element (IRP/IRE) system. IRP recognize motifs known as IRE, which are loops that are in the 3’ and 5’ untranslatable regions of the mRNA. Binding of the IRP to the IRE occurs when there are low levels of iron in the cell, and depending on whether it is located in the 5’ or in the 3’ end, it will either prevent the translation machinery from binding or increase its stability (preventing its degradation), respectively (87). The IRP/IRE system regulates the expression of mRNAs coding for proteins related to different aspects of iron metabolism (internalization, storage, heme group synthesis, export), such as TfR, ferritin or FPN. However, in recent years, a multitude of mRNAs with IRE sequences have been described in many genes such as the tricarboxylic acid cycle (TCA) aconitase enzyme (ACO2) or hypoxia inducible factor (HIF), among others (88, 89). This broadens the regulatory functions of the IRP/IRE system and provides evidence of its physiological action what expands beyond the direct control of the cell’s iron status and about how iron connects to other target signaling pathways that would allow the cell to adapt to challenging environments, such as hypoxia, inflammation, or infection.

Therefore, given that iron is essential for the activity of many proteins and is also involved in the expression of many other genes by the IRP/IRE system, manipulation of iron homeostasis in a cell as plastic as the macrophage would significantly affect its function. Besides that, macrophages modify their phenotype to adapt their iron metabolism to specific situations (M1/M2), such as an infection.

There are numerous differences between M1 and M2 macrophages, including the pattern of secreted cytokines, the type of energy metabolism, and surface markers. Additionally, iron metabolism is also differentially regulated in macrophage polarization (90), leading to differences in intracellular and extracellular iron levels, which are appropriated to their specific function. It is estimated that about 60% of genes related to iron homeostasis are differentially expressed in late stages of macrophage polarization (91). In general, M1 macrophages have a phenotype of ferritin^high^, TfR^high^ and FPN^low^, so they tend to retain iron and remove it from the exterior, which has been shown to enhance their antimicrobial effector functions such as ROS production. In contrast, M2 macrophages show the opposite profile (ferritin^low^, TfR^low^ and FPN^high^), being cells that tend to export iron, which is linked to their immunoregulatory and tissue repair functions (92, 93).

If questioning the impact of iron on macrophage polarization, results are not always in accordance. Polarization pathways are complex, and it has been shown that iron can modulate the macrophage phenotype at different levels (signaling, metabolism, and epigenetics) [reviewed in (94)]. Although the effects of iron on the macrophage depend, among other things, on the iron source, concentration, exposure time and cellular context, studies generally show that iron supplementation induces a M1-like phenotype with pro-inflammatory cytokine production and ROS generation (90, 95–100). For example, a close relationship between NOS (M1 marker) and iron has been described. In addition to being an iron-requiring hemoprotein at its catalytic center, iron levels in the macrophage have been shown to control the transcriptional expression of the enzyme, and NO, in turn, modulates the expression of genes related to iron metabolism by promoting increased binding of IRP to IRE motifs (101–103).

On the contrary, and in general, iron deficiency has been shown to limit pro-inflammatory phenotype activation and promote an anti-inflammatory phenotype (104–106). In this regard, iron deprivation in macrophages by altering TCA has been shown to decrease the inflammatory response upon exposure to lipopolysaccharide (LPS) (107). This metabolic shift has been linked to iron acting as a cofactor for several mitochondrial electron transport chain complexes, hindering OXPHOS. Moreover, iron deprivation has been associated with reduced expression of proteins such as succinate dehydrogenase complex iron sulfur subunit B (SDHB), a subunit of the mitochondrial complex II, as its mRNA contains IRE motifs (106, 108).

Collectively, these findings suggest that iron levels within macrophages can elicit diverse immunomodulatory effects through the regulation of multiple signaling pathways, and vice versa, the polarization state of the macrophage determines its iron content to match its metabolism to its function. Thus, manipulation of iron levels has been proposed as a promising tool to modulate the polarization state of macrophages. With this strategy, and more specifically, with the administration of iron in the form of IONPs, macrophages have been re-educated in different scenarios, achieving significant advances in the field of cancer and infectious diseases.

### 3.2 *Leishmania* and macrophages: two organisms with the same goal, iron

Infecting a host provides pathogens with access to a nutrient-rich environment. Thus, hosts employ strategies to hinder microbial access to these resources, a phenomenon known as nutritional immunity (109). In the case of *Leishmania*, which is heme auxotroph and lack obvious iron storage systems, scavenging for host iron is an essential adaptation for survival and virulence (110).

In the membrane of phagolysosome there are transporters such as natural resistance-associated macrophage protein-1 (NRAMP-1) that are responsible for transporting iron to the cytoplasm, limiting the availability of iron to the pathogen (82). In fact, some of the determinants of susceptibility to leishmaniasis are certain polymorphisms in the NRAMP-1 transporter, reflecting the competition between the intracellular form of *Leishmania* and the host for the small amount of iron that enters the phagolysosome (111, 112). To counteract this access limitation to iron, *Leishmania* has evolved a multitude of strategies throughout its evolution to provide itself with the metal. For example, it has been described that *L. donovani* secretes a tryparedoxin peroxidase (TXNP), which downregulates NRAMP-1 activity and prevents iron levels in the phagolysosome from declining (113).

*Leishmania* tries to redirect the iron that reaches the macrophage to its cellular niche by altering iron trafficking pathways. Studies indicate that, when macrophages are infected with *L. amazonensis*, there is a remarkable fusion of endocytic vesicles containing transferrin bound to its receptor with the phagolysosome, delivering iron to the parasite (114). Additionally, various *Leishmania* species hinder the expression of FPN to inhibit the metal exit from the cell and increase its availability for incorporation into their own metabolism (115, 116). Attempts have been made to counteract this strategy by administering FPN-loaded NPs to *L. major*-infected mice, reducing the parasite load successfully (117).

*Leishmania* is also capable of altering and lowering the LIP, which is known to be crucial as an indicator of cellular iron demand as it is the fraction of metabolically available iron. When LIP levels are low, it indicates that the cell needs more iron. In this sense, *L. donovani* can deplete the iron pool to activate cellular iron sensors, triggering a response that increases the intracellular concentration of available iron. By inducing this response, the parasite gains access to more iron, which promotes its growth (118). Recently, it has been reported that *L. donovani* is also able to increase the fraction of available iron by cleaving poly(rC)-binding proteins (PCBPs), which are ferritin chaperones. What happens is that, as *Leishmania* degrades PCBPs, the loading of iron with ferritin is impeded, and the iron, instead of being stored in complexes, accumulates thus rendering available to enhance growth (119).

In addition to trying to maintain ferric levels both in the cytosol and within the phagolysosome, *Leishmania* must possess an iron transport system on its membrane in order to internalize this essential nutrient, as there is no evidence that trypanosomatids express siderophores like bacteria (120). First, since most of the iron within the parasitophorous vacuole is in its oxidated form (Fe^3+^), it needs ferric reductases to convert it to the soluble Fe^2+^ form, which can cross its membrane. Thus, *Leishmania* expresses the *Leishmania* ferric iron reductase 1 (LFR1) in their membrane and it is essential for its virulence. Without it, no matter how much iron is available, that it would not be able to use it (121, 122). The reduced iron is then transported into the parasite via the *Leishmania* Iron Transporter (LIT1). Interestingly, mutants lacking LIT1 can survive if LFR1 is overexpressed, suggesting the presence of alternative, lower-affinity iron transporters (120).

*Leishmania* also has transporters that allow it to internalize iron in the form of the heme group, which is already part of a multitude of enzymes in its metabolism and lacks the biosynthetic pathways of the heme group. Thus, it expresses *Leishmania* heme response 1 (LHR1) and the recently discovered *Leishmania* feline leukemia virus subgroup C receptor (LFLVCR), which is responsible for importing heme into its cytoplasm (123, 124). Moreover, *Leishmania* also expresses a hemoglobin receptor (HbR), as an alternative route for the acquisition of the heme group (125).

### 3.3 Iron, ally or enemy: the therapeutic use of iron in *Leishmania* infection

Competition between pathogens and hosts involves different war fronts, and the nutritional struggle plays a central role. Similar to what the host tries to do, and since iron availability is critical for the growth of *Leishmania*, the development of drugs aimed to limit access of iron to the parasite could be a strategy to control the infection. In this regard, different chelating agents have been tested to decrease the amount of iron accessible to *Leishmania* (126–131)(**Table 1**). For example, the use of quercetin as well as caffeic and rosmarinic acids, with described iron chelating activity, have been found to exert a leishmanicidal effect in different models (126–129). Furthermore, in mice infected with VL-causing *L. chagasi* and treated with desferrioxamine (DFO), a significant reduction of parasite load in the spleen and liver is observed after six weeks of infection (130).

**Table 1:** Current studies on iron manipulation as a host-directed therapy against leishmaniasis.

Type of strategy	Iron source/ Chelating agent	Parasite strain	Model	Dosage regimen/ concentration	The most relevant effects described	Reference
**Iron overload**	FAC	*L. major*	*In vivo* BALB/c Footpad	2 mg/kg/day Oral administration 4 times/week (10 weeks post-infection)	The parasite deregulates iron homeostasis locally and systemically. FAC treatment limits the progression of infection by triggering ROS production in the footpad.	(132)
**Iron depletion**	Quercetin	*L. braziliensis*	*In vitro* Peritoneal macrophages	48-70 μM	A decrease in the labile iron pool and in the infection rate was shown after quercetin treatment. The effect was not dependent on ROS or RNS production.	(127)
		*L. braziliensis*	*In vivo* Hamster	20 mg/kg 8 weeks, 5 times a week	The treatment reduced the lesion thickness and parasite load in the infected hamsters.	(126)
	DFO	*L. major*	*In vivo* BALB/c	10 mg/mouse/day 10 days	DFO produced a transient delay in the development of cutaneous lesions	(131)
		*L. chagasi*	*In vivo* BALB/c	10 mg/mouse/day 3 doses per week	DFO treatment caused a sharp drop in hemoglobin levels and a reduction in parasite load in the liver and spleen. No changes in cytokine profile were observed.	(130)
	CA	*L. amazonensis*	*In vitro* Peritoneal macrophages	12.5-50 μg/mL	CA decreased the infection rate by increasing levels of TNF-α, ROS, and NO, and simultaneously decreasing IL-10 levels and iron availability.	(129)
	RA	*L. donovani*	*In vitro* RAW 264.7	3.12–50 μg/mL	RA inhibited the growth of intracellular amastigotes and decreased the availability of iron.	(128)

FAC, Ferric ammonium citrate; DFO, Desferoxamine; CA, Caffeic acid; RA, Rosmarinic acid; TNF-α, Tumor necrosis factor-alpha; ROS, Reactive oxygen species; NO, Nitric oxide.

However, it seems that there is a critical point at which the parasite manages to overcome this limitation to iron access, perhaps by up-regulating the systems it has developed in this parasite-host evolution, and the deposit of iron present in tissues is enough to sustain its growth. Thus, Bisti et al. found that DFO treatment of *L. major*-infected mice resulted in a delay in skin lesion development, but after about 12 weeks the lesion sizes were similar between the control and treated groups (131). Comparably, dietary iron restriction in mice, although significantly decreasing iron levels in the liver and spleen, did not affect the growth of *L. infantum* (133).

Thus, we suggest that the strategy of treating with iron may be more beneficial in this infective context, since, as we have described, iron can modulate the macrophage phenotype, enhancing the host defense response (**Table 1**). In contrast to other pathogens, where iron overload is associated with increased susceptibility, iron administration in murine models of CL limits parasite growth (132). Banerjee and Datta observed that infection with *L. major* in the footpad caused an increase in iron in the infected area while causing anemia-like symptoms (low systemic iron and hemoglobin levels). These observations prompted them to treat mice with ferric ammonium citrate (FAC), which resulted in infection control and restoration of iron balance at systemic level. The iron supplementation strategy was successful in restricting parasite growth by promoting a local oxidative response (ROS generation) (132).

Interestingly, Charleboir et al. studied the response of hemojuvelin (HJV) knockout mice to infection with *L. major* and *L. infantum* (134). HJV is a membrane receptor involved in regulating the expression of hepcidin - a central hormone to the regulation for iron metabolism regulation. Mutations in the gene coding for HJV correlate with low hepcidin expression and severe systemic iron overload, known as hemochromatosis (135). The reason is that hepcidin is responsible for controlling the efflux of iron at cellular level by FPN, promoting its internalization and degradation. In cases of low hepcidin levels, this regulation is absent, and when iron enters the cell, it is exported to the bloodstream via FPN, generating a pathological state with iron excess (136).

In HJV knockout mice, tissue macrophages overexpress FPN on their surface, so they are unable to retain iron and release all of it into the plasma (134). Therefore, unlike the iron overload model generated by FAC administration, in a situation of hemochromatosis, tissue iron levels are low, and macrophages become extremely iron depleted. All this means that, when infected mice with *L. infantum*, there is no difference in disease progression or parasite load in the spleen and liver between mutant mice and wild-type controls. However, in the case of CL, like what happened with chelating agents, although there is a delay in the development of skin involvement compared to wildtype controls, at longer times, wound sizes are similar (134).

Therefore, since *Leishmania* is an intracellular parasite, we concur with several authors who emphasize the importance of localizing iron overload within macrophages, where the battle between the immune system and the parasite takes place. One way to ensure that iron reaches macrophages is to deliver it via NPs.

## 4 Nanoparticles to increase drug-*Leishmania* confluency. Activation and reprogramming of macrophages with iron oxide nanoparticles

A major hurdle to overcome with current leishmanicidal drugs is their low specificity/selectivity for macrophages, causing them to be distributed throughout the body. This, coupled with the fact that they are often cytotoxic, leads to many adverse effects for patients. Therefore, there is a clear need to improve the delivery of anti-leishmanial molecules to their site of action (the macrophage) to enhance their efficacy while decreasing their toxicity, being nanomedicine the right tool to fulfill this purpose. The selective targeted drug delivery to the site of action within the body without affecting healthy organs and tissues results in improved efficacy and lower side effects (137).

Nanomedicine has other advantages: protecting the drug from rapid degradation in the organism, improving drug stability, ability to cross biological barriers and reach specific intracellular compartments within the site of action, and capacity to control drug release (138). Nanomedicine makes use of NPs, which are defined as particulate dispersions or solid particles with a size in the range of 10-1000 nm (139). The drug can be incorporated into the NP matrix by encapsulation, adsorption to its surface or covalent conjugation. There are NPs of very different composition and morphology, organic (polymeric, lipidic) and inorganic (gold, silica, iron oxides) (140).

The propensity of macrophages for phagocytic clearance offers a perfect scenario for diseases where, like leishmaniasis, macrophages play a central role (141). In essence, since both *Leishmania* parasites and NPs share a common fate, the use of nanosized delivery systems would enhance the convergence of parasites and drugs, thereby reducing their toxicity. Indeed, the introduction of a liposomal amphotericin B formulation (AmBisome^®^, Gilead Sciences) marked a significant advancement in the treatment of VL as the first nanomedicine to enter the market. This clinical success is justified because both VL parasites and AmBisome^®^ are preferentially uptaken and accumulated in macrophages of the liver, spleen, and bone marrow. Moreover, AmBisome^®^ creates a depot inside macrophages that slowly releases the drug in a way that avoids its hemolytic effects and nephrotoxicity (142, 143).

**Figure 4:**
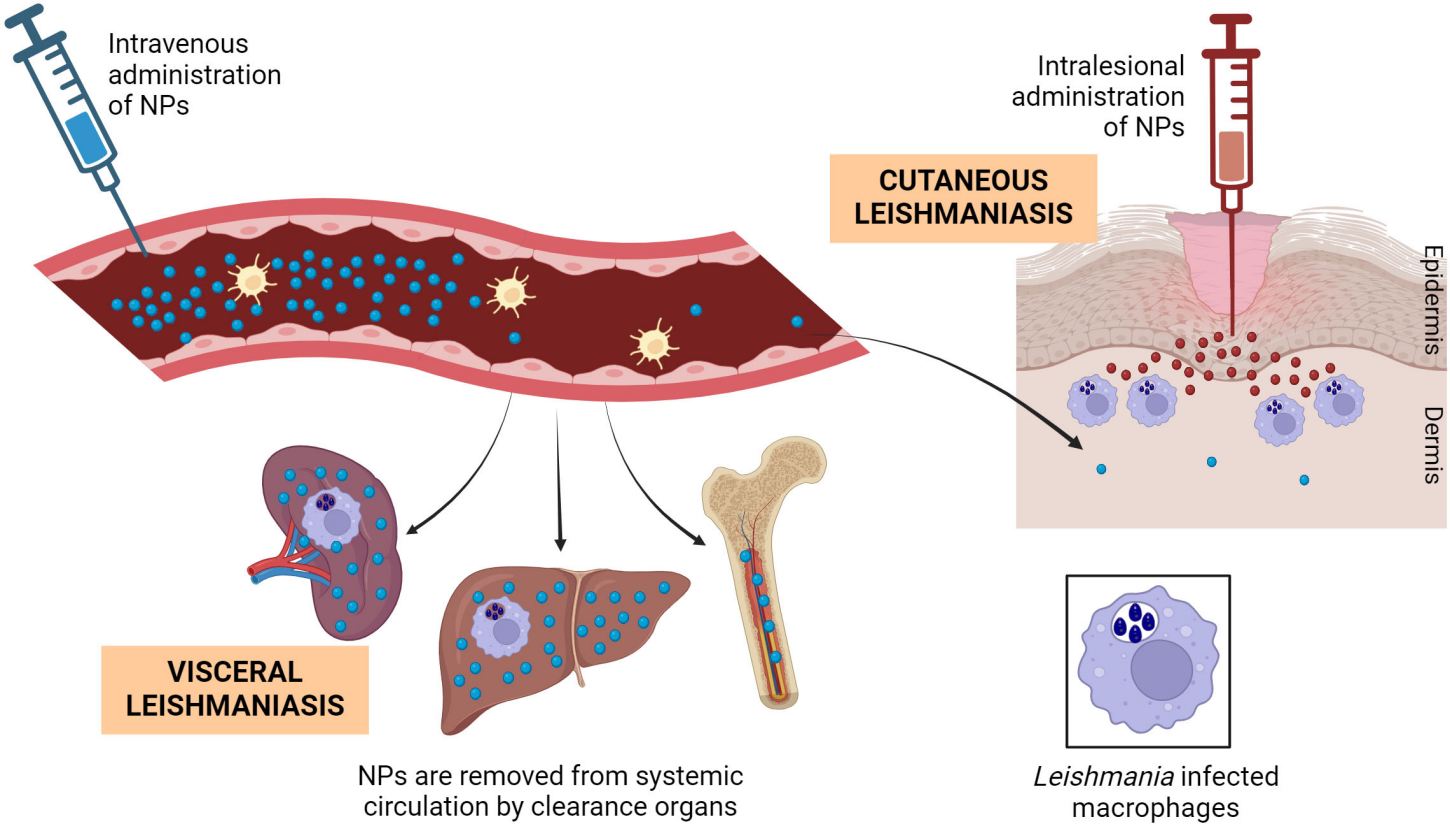
Biodistribution of NPs and encounter with *Leishmania-*infected macrophages. After their intravenous administration, NPs in general tend to accumulate in the macrophages of organs with fenestrated vasculature such as liver, spleen and bone marrow, also the major *Leishmania* hosts in VL. Only a small fraction of long-circulating NPs will get *Leishmania*-infected skin lesions. After topical administration and even in damaged skin, very small NPs have poor chance of arriving the dermal infected macrophages, making mandatory their intralesional (and uncomfortable) administration. Figure created with biorender.com.

However, it is essential to recognize that while macrophages are the target cells in all clinical manifestations of leishmaniasis, the affected organs may vary. In VL, the spleen and liver serve as the primary affected organs, providing an ideal environment for NP treatment due to their role as main clearance organs (**Figure 4**). Nonetheless, addressing macrophages specifically in various CL forms poses challenges, as lesions are often distal and deep-seated in the skin, making the delivery of therapeutic agents difficult, whether locally or systemically (144). Indeed, AmBisome^®^, the first-line treatment for VL, has demonstrated reduced efficacy in treating cutaneous forms of leishmaniasis, likely because parasites primarily localize in dermal macrophages near the sandfly bite and draining lymph nodes (145). Inflammation at this site increases vascular permeability, allowing a fraction of liposomes to extravasate. However, the extravasated liposomes constitute a lower proportion of the total administered dose (146).

To enhance the delivery of NPs to inflammation sites in CL, exploring alternative administration routes and tailoring NPs properties to evade rapid clearance by liver, spleen, and bone marrow macrophages is crucial. Consequently, significant efforts are being made to improve the formulation of amphotericin B liposomes, including modulation of composition and size. Generally, smaller liposomes prolong circulation time, thereby increasing accumulation at the lesion. Additionally, pegylation and functionalization of NPs with various ligands have been explored as strategies to prevent opsonization by plasma proteins and enable specific recognition by targeting tissues and cells (147).

### 4.1 Iron oxide nanoparticles

IONPs are composed of iron and oxygen atoms, forming a distinctive core-shell structure. The core is typically made of iron oxide (maghemite γ-Fe_2_O_3_, magnetite Fe_3_O_4_, hematite α-Fe_2_O_3_ and goethite FeO(OH)), while the shell can be functionalized with diverse compounds such as small molecules, metal ions, surfactants, and polymers (148). This unique composition grants IONPs remarkable properties that have found utility across various fields, including medicine, electronics, and environmental remediation (148–150).

In medicine, IONPs have garnered attention for their applications in diagnostics and therapies. They serve as contrast agents in magnetic resonance imaging (MRI), providing intricate details of tissues and disease conditions. Moreover, their versatility extends to drug delivery, where they have shown success in targeted delivery systems, augmenting treatment precision and efficacy while reducing adverse effects (17). Additionally, IONPs have been effectively utilized to induce photothermal or magnetic local hyperthermia, further expanding their therapeutic potential (151). Notably, the FDA has approved certain IONP-based products. For instance, Feridex^®^, which is composed of dextran-coated IONPs (ferumoxide), or Resovist^®^ (ferucarbotran), comprised of IONPs coated with carboxydextran, are approved as imaging contrast agents for the detection of liver lesions. Another FDA-approved IONPs, Feraheme^®^ (ferumoxytol), an IONP-based drug, has been sanctioned for the treatment of iron deficiency anemia in patients with chronic kidney disease or patients who do not tolerate oral iron supplementation (17).

A concerning issue is the biodistribution of IONPs, which is influenced by various factors such as their size, shape, surface coating, and charge, as well as the dose and route of administration [reviewed in (152)]. When administered intravenously, IONPs have a half-life ranging from 40 minutes to 24 hours and tend to accumulate in macrophages of the liver and spleen. However, when these organs are saturated, IONPs can also be distributed to other tissues such as the lungs, kidneys, and heart. Regarding their excretion, it is suggested that larger IONPs are eliminated via feces, while smaller ones are primarily excreted by the kidneys through urine (153, 154).

In addition, the effect of IONPs at cellular level will depend directly on their degradation by the cell, i.e. the release of the iron product. This contrasts with the administration of iron in salt form, where the effect is more rapid due to the instantaneous availability of iron ions for cellular use. Thus, within macrophages, IONPs enter via the endocytic pathway and accumulate in lysosomes. Here, the acidic pH triggers their degradation and subsequent release of iron into the cytoplasm. This delay in iron availability and cell use probably explains why, *in vitro*, iron in the form of salt can metabolically activate macrophages more than IONPs within 24-72 hours at any concentration (155). In addition, the behavior of IONPs compared to iron salts is significantly different in both *in vivo* and *in vitro* models, with salts exhibiting greater toxicity. This discrepancy is influenced by factors such as stability and aggregation capacity (155, 156).

### 4.2 IONPs pull the rope: macrophages reprogramming in cancer

Considering the intricate molecular and functional machinery that iron orchestrates in macrophages, IONPs have become a valuable tool to influence the immune response of this cell type. Thus, owing to their ability to accumulate preferentially in macrophages, IONPs offer promising prospects for novel therapeutic strategies, intending to harness the immune system’s strength to combat diseases such as cancer or infections.

In the field of cancer treatment, IONPs have been used to immunologically modulate the tumor microenvironment, in particular macrophages (157). Recent studies have outlined that utilizing IONPs in cancer treatment holds the potential to diminish tumor cell growth by reprogramming or re-educating tumor-associated macrophages (TAMs) from an M2-like pro-tumoral into an M1-like anti-tumoral phenotype.

For example, the FDA-approved IONP compound ferumoxytol significantly inhibited the growth of subcutaneous adenocarcinomas and prevented hepatic metastasis in mice. The effect was be accompanied by increased presence of pro-inflammatory M1 macrophages that attacked cancer cells in tumor tissues (158). Since then, the antitumor effect of ferumoxytol (alone or in combination with other immunomodulatory drugs) has also been reported for other types of cancer in *in vivo* models (159–162).

In addition to ferumoxytol, alternative types of IONPs have been investigated as potential antitumoral agents. For instance, NPs referred to as cross-linked iron oxide (CLIO) or IONPs coated with an anti-CD206 antibody (for targeting M2 macrophages) have demonstrated the capacity to attenuate tumor dimensions through their influence on macrophage polarization (163, 164). To augment this effect on the polarization of TAMs, Wu et al. loaded IONPs with L-arginine, with the aim of elevating NO secretion to induce tumor cell death. This NO-centered therapy was evaluated in a breast cancer model, revealing heightened NO levels and increased M1 markers, accompanied by an apoptotic antitumoral impact (165).

Following an innovative approach, Li et al. (166) employed IONPs to reprogram macrophages *in vitro* and subsequently used them as a therapeutic product, a strategy known as cellular therapy. In this study, murine RAW 246.7 macrophages were treated with IONPs coated with hyaluronic acid. After confirming their polarization into a pro-inflammatory phenotype and the enhancement of their innate functions (ROS and cytokine production), these modified macrophages were administered to mice bearing 4T1 tumors, resulting in a significant suppression of tumor growth. Remarkably, in addition to their cancer cell elimination abilities, the introduced macrophages polarized resident M2 tumor-associated macrophages into a M1-like phenotype in a paracrine-like manner. Moreover, the administered macrophages were guided to the tumor site using a magnetic field, leading to increased accumulation and a more pronounced antitumoral effect (166).

It’s essential to consider that, while IONPs have the ability to polarize macrophages towards an M1 phenotype in a tumor context, under certain circumstances, they can inhibit this inflammatory response by inducing polarization towards an M2 phenotype (167–169). Indeed, several studies have shown that when macrophages are exposed to IONPs together with LPS, a change in the pattern of gene expression occurs, with an up-regulation of M2-related genes and a reduction in the secretion of NO (170, 171). This is explained by the competition and regulation of the TLR4 receptor, the target of both LPS and IONPs. This difference in the phenotype induced by IONPs on macrophages highlights the complexity of the process, which is dependent on system composition, shape, size, coating and dose administered. However, in general, it can be concluded that in most cases and independently of their physicochemical characteristics, IONPs promote an M1 phenotype in macrophages, both in tumor and non-tumor tissues, even in the presence of a pro-M2 stimulus such as IL-4 (157).

### 4.3 How do IONPs manage to reprogram macrophages? A tangled skein of interactions

IONPs exert a crucial role in modulating the immunological functions of macrophages. Indeed, exposure of primary mouse macrophages to such naked IONPs has identified more than 1,000 differentially expressed genes compared to untreated controls, most of them linked to inflammatory response and oxidative stress (172). This wide variation in gene expression underscores the complexity of the biological responses triggered by the interaction between particles and macrophages and raises the challenge of understanding which cell signaling pathways orchestrate this enormous effect on transcription.

In general, and as we have seen in some examples within in a tumor context, there is evidence that IONPs promote an M1 profile in macrophages, with increased expression of M1 markers and decreased expression of M2 markers (173). However, the mechanisms of how IONPs favor a pro-inflammatory phenotype are not uniform. Until now, the molecular basis of IONPs and macrophage interactions have remained enigmatic due to the heterogeneous chemical composition and diverse physical properties of NPs (size, charge, and morphology), as well as the array of *in vitro* and *in vivo* models employed.

The first step in the IONPs-macrophage relationship is their interaction and internalization, which depends on the coating of the particle and also on the so-called protein corona complex (PC) that forms on the surface of the particle when in contact with biological fluids. The composition of the PC critically affects the interaction with the macrophage, and a recent proteomic study has shown that its composition depends more on the origin of the biological fluid (animal species) than on the coating of the particle (169). Furthermore, they studied whether this difference in PC composition affects its ability to modulate the macrophage phenotype *in vitro*. Interestingly, in the case of IONPs coated with dimercaptosuccinic acid (DMSA), they promoted in RAW264.7 macrophages a phenotype more similar to M2 when using a medium supplemented with mouse serum and a phenotype closer to M1 when using fetal bovine serum, tangling the skein a little more (169).

It is believed that IONPs interact with TLRs on the macrophage surface, primarily with TLR4. Clinically approved IONPs (ferucarbotran and ferumoxytol) have been shown to stimulate the production of pro-inflammatory cytokines (IL-1β, IL-12, TNF-α, IL-2, and IL-10) by binding to and activating TLR4 (174). Furthermore, pretreatment of macrophages with CLI-095 (a TLR4 inhibitor) prior to IONPs treatment significantly reduces the expression and levels of secreted pro-inflammatory cytokines. This implies that induction of the M1 phenotype is hindered by TLR4 blockade, highlighting its relevant role in macrophage interaction and activation (174, 175).

Another discovery supporting the IONPs-TLR4 relationship is that while polyethylene glycol (PEG)-coated IONPs exhibit a pro-inflammatory effect on macrophages per se, co-exposure of these particles with LPS attenuates the inflammatory response typically induced by this bacterial molecule. Since LPS is recognized by TLR4, it suggests that IONPs might compete for binding to the same receptor as LPS, thus blocking its binding and limiting its stronger pro-inflammatory effect (170). In addition to the central role of TLR4, small IONPs (10-60 nm) also interact and activate other surface TLRs such as TLR2 and TLR6, as well as the intracellular TLR8 (176, 177).

**Figure 5:**
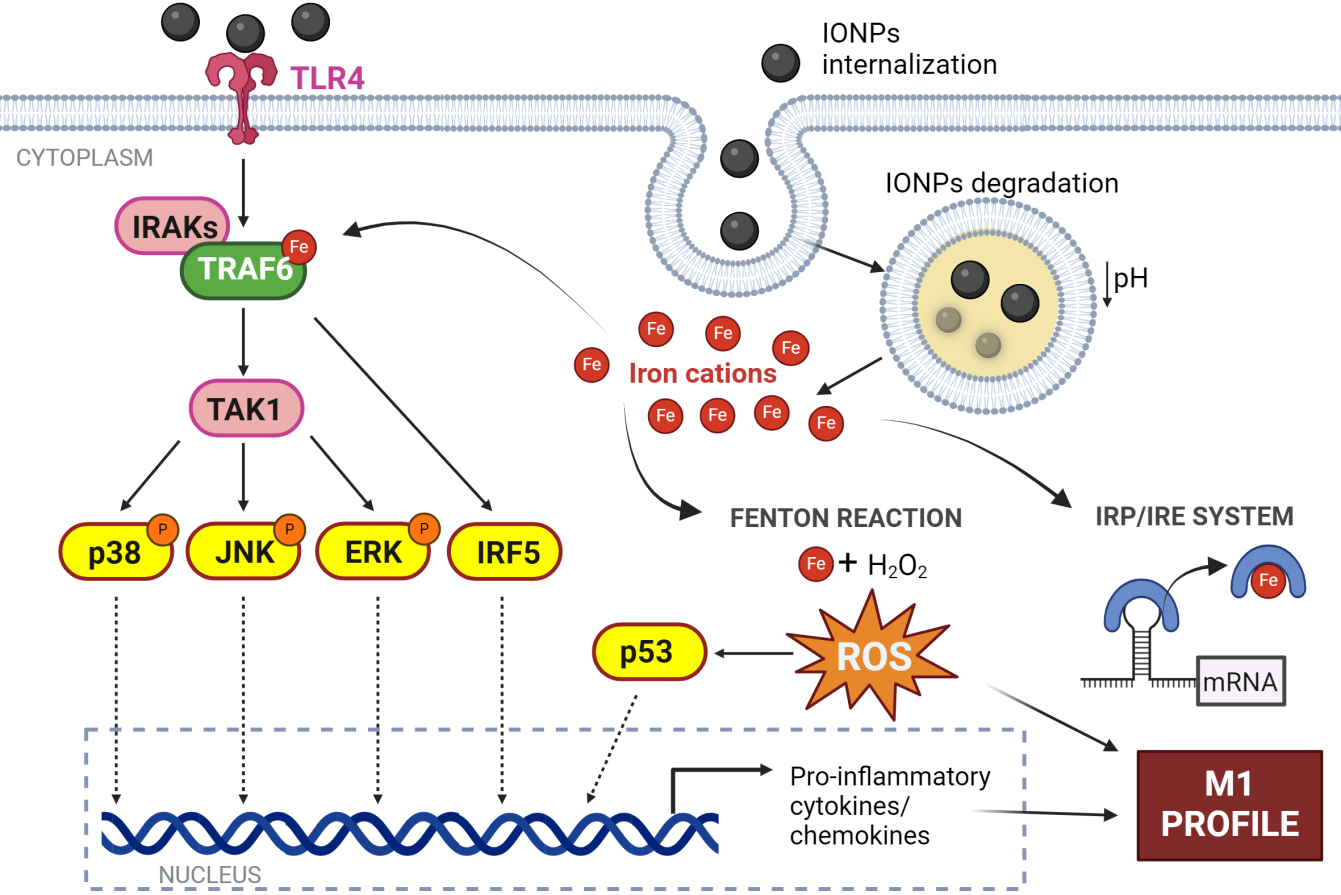
Effect of IONPs on macrophages. Interaction of IONPs with TLR4 initiates the activation of IRF5 and several MAPKs culminating in the expression of inflammatory response genes. In addition, IONPs are internalized by macrophages and are biodegraded in the phagolysosome resulting in the release of iron cations into the cytosol. This free iron induces ROS production by the Fenton reaction; and modulates protein activity by acting as a cofactor, and protein expression by the IRE system. All this converges in the induction of an M1 profile in the macrophage. Figure created with biorender.com.

Upon binding of IONPs to TLRs, particularly TLR4, a cascade of intracellular signaling is triggered (**Figure 5**). The interaction of macrophages with IONPs has been associated with the activation of various signaling molecules within the canonical TLR pathway. Notably, the impact of IONPs on the ubiquitination of TNF receptor-associated factor 6 (TRAF6), a key adaptor protein in mediating TLR signal transduction, has been confirmed. TRAF6’s autoubiquitination is essential for subsequent steps in the signaling cascade and is facilitated by the presence of iron (178). In *in vitro* experiments involving macrophages, co-localization of IONPs with TRAF6 and ubiquitin was observed (179). Increased ubiquitination of TRAF6 leads to elevated interferon regulatory factor 5 (IRF5) expression, which has been linked to the induction of a more pronounced M1 phenotype in macrophages upon exposure to IONPs (179, 180).

TRAF6 acts as a platform for the downstream activation of several protein kinases such as the IkappaB kinase (IKK) complex or different members of the mitogen-activated protein kinases family (MAPK), pathways that lead to the activation of transcription factors linked to inflammatory responses and immune activation (181). Thus, numerous articles have described that the secretion of pro-inflammatory cytokines as a result of this IONPs-TLR4 interaction is dependent on the phosphorylation of p38/JNK/ERK kinases (174, 182–185) and subsequent activation of transcription factors such as NF-κB, AP-1, or the aforementioned IRF5 (159, 179, 186) (**Figure 5**).

IONPs not only exert their effect on the macrophage through interaction with cell surface receptors but are also efficiently taken up by macrophages as cells specialized in engulfing foreign particles. Different mechanisms of internalization of IONPs at cellular level have been proposed, such as clathrin-mediated endocytosis, caveolae-mediated endocytosis or receptor-mediated endocytosis, among others (169, 187–189).

Once inside the macrophage, IONPs localize to endocytic vesicles, which fuse with lysosomes (**Figure 5**). Exposure to this acidic environment (pH~5) induces their degradation and the release of iron cations from the NP core (183, 190). This free iron stimulates the production of ROS by the Fenton reaction leading to exacerbated oxidative stress. It has been shown that macrophages produce ROS in a concentration-dependent manner depending on the concentration of IONPs to which they are exposed (191, 192). This ROS generation resulting from treatment with IONPs has been linked to the overexpression of the p53 protein (193). This protein plays a crucial role in the regulation of ferroptosis, a type of cell death that depends on iron concentration (194). Thus, the presence of high levels of iron within the macrophage promotes the acetylation and subsequent activation of p53, which has been linked to the establishment of the M1 response (96). In addition, this free iron can modulate the expression of genes regulated under the IRE system (185) and the activity of numerous proteins that use it as a cofactor such as TRAF6, as we have described (**Figure 5**).

In short, IONPs trigger a complex and intricate network of molecular signaling in macrophages, a puzzle with missing pieces that is essential to understanding their impact on immune function.

### 4.4 IONPs against leishmaniasis: a promising therapy

The remarkable versatility of iron in modulating the effector functions of macrophages situates this metal as a promising weapon against leishmaniasis. In this context, IONPs offer a means to achieve targeted iron overload in macrophages, a vital aspect given that both co-localization with the parasite and appropriate concentrations are crucial for their effectiveness. The diversity in their application, with distinct sizes, charges, and coatings, reflects the adaptability of IONPs as nanotechnological weapons against leishmaniasis, although difficulties to draw conclusions on their use as a therapy. Some studies focus on their intrinsic leishmanicidal activity, while others use them as vehicles to administer specific drugs, employing IONPs as carriers of therapeutic agents to specific cells to enhance selectivity (**Table 2**).

**Table 2:** Studies on the application of iron oxide nanoparticles in the treatment of leishmaniasis.

Type of IONP	Size (nm)	Parasite strain	Model	Dosage regimen/concentration	The most relevant effects described	Ref
**Iron dextran**	NS	*L. major*	*In vivo* BALB/c mice Footpad	Peritoneal injection 4-8 mg/mouse/day 10 days. Before and after infection	Treatment limited the growth of the footpad thickness; upregulated IFN-γ and iNOS and decreased IL-4 and IL-10 expression; switched Ig isotype to IgG2a in sera	(131)
	NS		*In vivo* BALB/c mice Ear	Peritoneal injection 4 mg/mouse/day 10 days. Before and after infection	Iron-dextran limited the severity of cutaneous lesions. The effect was reverted by DPI treatment (decreasing the oxidative burst) and depended on the initial parasite dose.	(195)
					Iron-dextran contained infection in mice via NF-κβ TF activation; induced a phenotype resistant to reinoculation with promastigotes (maintained oxidative burst); increased CD4+ T-cell recruitment.	(196)
	NS	*L. infantum*	*In vivo* C57BL/6 BALB/c Mice	Peritoneal injection A single injection of 10 mg of iron/mouse. Before infection	Iron-dextran decreased the growth of the parasite in the liver and spleen; which was coupled with an oxidative burst. The effect was greater when iron accumulation occurs before infection	(133)
**PEI_25_ decorated *-γ-*Fe_2_O_3_ NPs**	40-50 nm	*L. major* *L. donovani*	*In vitro* THP1/J774 macrophages	0.3-0.5 μg/mL	IONPs exhibited cytolytic activity against various species of *Leishmania*, resulting from the rupture of lysosomes. Additionally, dermoscopic images revealed that the lesions in treated mice were less profound than those in untreated group	(197)
			*In vivo* Mice	Topical. Commercial cream with 0.067% w/w of iron Once a day for 10 days		
**Citric acid-coated Fe_3_O_4_ NPs**	66 nm	*L. mexicana*	Axenic amastigotes	200 μg/mL	Amastigotes treated with IONPs and then exposed to a magnetic field resulted in their death	(198)
**Piroctone olamine coated Fe_3_O_4_NPs**	15-20 nm	*L. major*	*In vitro* J774 macrophages	10-200 μg/mL	30 days after the start of treatment, the diameter of lesions in mice was reduced by less than half in both PO-coated and bare NPs. The effect was concentration-dependent and more pronounced in the coated ones. *In vitro* they promote NO production.	(199)
			*In vivo* BALB/c mice Base of the tail	Topical 1-2 mg/kg/day Once a day for 4 weeks		
**Ferromagnetic iron oxide nanorods**	116 nm	*L. tropica*	*In vitro* Peritoneal macrophages	0.08-10 μg/mL	Significant inhibition of amastigotes growth was observed even at low concentrations of the nanorods. The effect was enhanced by exposing macrophages to UV light, further stimulating ROS production	(200)
**IONPs synthesized via a green route**	4 nm	*L. amazonensis*	*In vitro* Peritoneal and RAW 264.7 macrophages	1-100 μg/mL	IONPs are distributed throughout the macrophage cytosol, reaching the interior of the PV and amastigotes. A marked anti-proliferative effect on amastigotes was observed	(201)
**Fe_3_O_4_ -bioMOFs nanocomposite**	> 100 nm	*L. major*	*In vitro* J774 macrophages	6.25-100 μg/mL	Treatment with the nanocomposite reduced the size of lesions in mice and induced a significant increase in IFN-γ and a decrease in IL-4 secretion from spleen lymphocyte	(202)
			*In vivo* BALB/c mice Base of the tail	Topical. Ointment with 25 ug/mL of IONPs. 3 times a week		

NS, Not specified; PO, Piroctone olamine; DPI ,Diphenyleneiodonium; PEI, Polyethyleneimine; MOF, Metal–organic framework; PV, Parasitophorous vacuole; iNOS, Inducible nitric oxide synthetase; NF-κB, Nuclear factor kappa-light-chain-enhancer of activated B cells; IFN-γ, Interferon-gamma; IgG, Immunoglobulin G; ROS, Reactive oxygen species; UV, Ultraviolet.

The initial investigations involving IONPs in the context of leishmaniasis were conducted by Bisti et al. in the early years of this century (131, 195, 196). They employed the topical application of IONPs-dextran as therapy in murine models of CL. Subsequently, this type of colloidal system has also been applied in VL models (133). In both scenarios, the leishmanicidal effect of iron relies on the localized generation of ROS and NO within macrophages, which are key effector molecules for infection control. Vale-Costa et al., compared the effect of iron administration in knockout mice for iNOS and for the p47^phox^ subunit of NOX2 with wildtype mice, and they found that parasite load falls was reduced in wildtype mice but not in mutants (133). Similarly in the CL model, Bisti et al. observed that the protective effect of iron overload was diminished when co-treating with diphenyleneiodonium (DPI), a NOX inhibitor (195). Furthermore, the same authors described that iron-mediated containment of infection was associated with an activation of NF-κB TF and the consequent pro-inflammatory response. Interestingly, they also observed increased recruitment of CD4+ T cells to the draining lymph node, inducing a sustained Th1 response and a protective state against *Leishmania*, as mice were resistant to reinfection with parasites 12 weeks after the first administration (196).

Abazari et al. also discussed the immunomodulatory potential of IONPs in the context of leishmaniasis. Following topical application of IONPs, there was an increase in IFN-γ secretion and a decrease in IL-4 levels in the lesions of infected mice, indicating the induction of a Th1 response that effectively controls the infection (202). As can be observed, all treatments based on IONPs for the cutaneous form of the disease have been tested through topical administration. It’s worth noting at this point that all formulations of IONPs that have been approved for clinical use are administered intravenously or orally (203). Regarding studies on skin penetration of IONPs, similar to other types of NPs, particles size and composition (flexibility) are determining parameters for their ability to penetrate into the skin as they enter primarily through the stratum corneum via the intercellular route. Thus, it has been observed that small IONPs ranging from 10-15 nm can penetrate the epidermis but are unable to reach the dermal layer (204–206), where infected macrophages are located. Despite the skin damage resulting from the infection, it has been demonstrated that no type of NPs can reach the areas where macrophages infected with *Leishmania* reside (207). Consequently, the proven leishmanicidal effect of IONPs in topical treatments suggests two possible mechanisms: either the free iron resulting from the degradation of the particles reaches the infected areas and act on these macrophages, or the key molecules for parasite control, such as ROS and NO, are generated in more superficial layers and diffuse towards the areas where the parasite is located.

Turning to the activity of IONPs, interestingly, the production of ROS/NO, which imparts their pro-inflammatory effect, can be amplified under exposure to light (photons). The photocatalytic prowess of IONPs is unveiled through light absorption, transferring energy to molecular oxygen and water, thereby boosting ROS production in the presence of light. Significantly, the study by Islam et al. with iron oxide nanorods (IONPs with elongated morphology) reveals an enhanced anti-amastigote effect of IONPs under exposure to LED light *in vitro* (200). Another leishmanicidal application of IONPs involves their use in photothermal therapy, generating heat when exposed to an alternating magnetic field. This technology has proven capable of raising the temperature in the NP-occupied area to 45°C, eliminating parasites effectively. Observations indicate success in killing axenic amastigotes of *L. mexicana* using this approach (198).

An intriguing finding is that IONPs seem, in some cases, to affect the survival of both promastigotes and amastigotes (199, 202, 208). However, recent research, exemplified by Vercoza et al., indicates that the effectiveness of IONPs is confined to the intracellular stage, emphasizing their immunomodulatory influence on macrophages rather than a direct impact on the parasite. The explanation does not stem from a variance in IONP penetration into promastigotes and amastigotes, as both life forms were observed to successfully accumulate these particles (201).

Among the studies using IONPs as a carrier, the vehiculization of AmB, the reference drug for VL, stands out. The use of IONPs as a vehicle in this context led to a two-fold reduction in parasite load compared to the administration of the drug alone, thus reaffirming its capacity for selective accumulation within infected macrophages (209). Concerning coatings, IONPs have been decorated, for example, with polyethyleneimine (PEI) and with pyroctone oleamine, for their proven cytolytic effect by embedding in cell membranes. In both cases, their leishmanicidal capacity has been tested both *in vitro* and *in vivo*, and the coatings enhance their efficacy to act compared to bare particles (197, 199). This duality of direct and vehicle strategies offers an immense range of possibilities to address the complexity of infection, maximizing therapeutic efficacy and selectivity of action against the parasite.

These findings highlight the multiple properties of IONPs, which make them a promising tool to revolutionize the treatment of leishmaniasis. Their versatile nature opens up diverse avenues for impressive therapeutic strategies, marking an important step forward in the search for effective and targeted interventions against this parasitic disease.

## 5 Limitations of IONPs as macrophage-directed therapies for treating leishmaniasis

Due to the ability of IONPs to selectively accumulate in macrophages, where *Leishmania* parasites live, and considering that intracellular iron balance is crucial for regulating macrophage polarization status, IONPs emerge as a promising strategy for treating leishmaniasis. This review has shown that IONP-challenged macrophages initiate a series of processes that trigger an activation towards an antimicrobial M1 phenotype (inflammatory response), capable of eliminating *Leishmania*. The proinflammatory effect would be mainly mediated by IONP interaction with TLR4 and iron overload.

**Figure 6:**
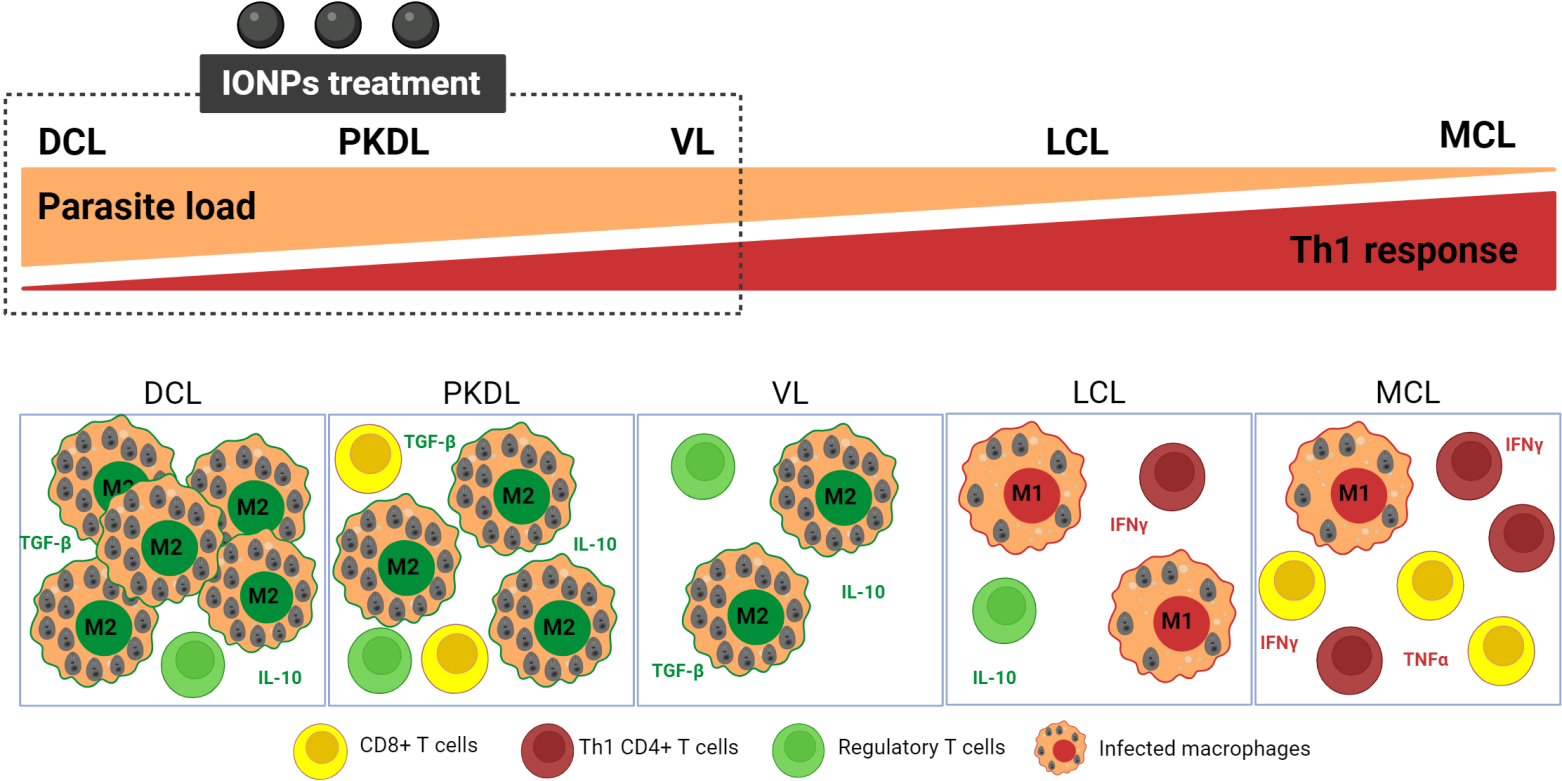
Scheme of immune profiles of the main clinical manifestations of leishmaniasis and suitability of IONPs. The balance between Th and macrophage responses highlights the suitability of IONP as a potential therapeutic strategy for VL, MCL, and PKDL, where macrophage abundance and/or pronounced T-Regulatory response are prominent features. DCL,Diffuse Cutaneous Leishmaniasis; PKDL, Post-Kala-Azar Dermal Leishmaniasis; VL, Visceral Leishmaniasis; LCL, Localized Cutaneous Leishmaniasis; MCL, Mucocutaneous Leishmaniasis. Figure created with biorender.com.

However, this strategy has limitations. Apart from the accessibility of IONP to infected macrophages (limited in the cutaneous lesions) either after parenteral or local administration, a questionable issue is the targeting of macrophages as an immunomodulatory approach for the treatment of leishmaniasis versus other immune cells. In fact, the targeting of TAM in cancer is well suitable for those types of tumors with a high proportion of these immune cells whose polarization decide the overall tumoral microenvironment. The situation could be similar in DCL (diffuse cutaneous leishmaniasis) and PKDL (post kala-azar dermal leishmaniasis). The skin lesions of these clinical manifestations are characterized by a high number of heavily M2-like infected macrophages due to a lack of Th1 response and high levels of TGF-β and IL-10 (Regulatory T-cells) (210). Th bias would be similar in VL although the macrophages would not be the most abundant immune cells. However, in MCL, there is a low proportion of poorly infected macrophages. IFN-γ and TNF-α producing Th1 cells and cytotoxic CD8+ cells are predominant while levels of IL-10 are low, leading to exacerbated inflammation and tissue destruction (**Figure 6**) (210). Thus, in this clinical manifestation, *Leishmania*-infected macrophages phenotype would most closely resemble M1-macrophages, despite which parasites survive (211). IONPs would be a suitable strategy for VL, DCL or PKDL. All of them would also benefit from the blockage of immune-checkpoints such as TGF-β, IL-10, programmed cell death protein 1 (PD-1), programmed cell death ligand 1 (PDL-1) or cytotoxic T lymphocyte antigen 4 (CTLA4) (212, 213). On the contrary, IONP would be even contraindicated as MCL immunotherapy (**Figure 6**). The most suitable for this clinical manifestation would be the combination between leishmanicidal drugs (for clearance of the parasite) with immunomodulatory strategies addressed to avoid tissue destruction (207), such as the inhibition of TNF-α (with pentoxifylline), the cytolytic activity (Tofacitinib) or inflammasome activation (214).

IONPs could also have an effect on T cell status and bias, although it should be indirectly produced by extracellular IONPs derived products (Fe or redox) or macrophage-mediated. The targeting of T cells with NPs is very limited by physiological barriers (position within organs) and the non-phagocytic nature of these cells, unlike macrophages, which are very well positioned for NP sampling and have very active and diverse endocytic pathways.

Another aspect to consider is the critical role that macrophage polarization status has not only the parasite clearance but also in tissue remodeling. In-depth, the persistence of M1-macrophages hampers the correct process of wound healing and tissue remodeling. Thus, the stimulus prone to produce M1-bias should be temporal and be removed after parasite clearance. In line with this, we cannot forget the dark side or immunotoxicity produced by IONPs mainly through their effect on oxidative stress and ongoing inflammation or cellular components alterations (215). Some IONPs such as Ferumoxide and Ferucarbotran initially approved in the USA and Europe for liver imaging were withdrawn from the market due to different toxic effects. Others such as Ferumoxytol remain approved for iron deficiency treatment for patients with chronic kidney disease and have recently been shown to be suitable for contrast aging (216). It is necessary a better comprehension of the relationships between IONP properties (iron release kinetics, stability, biodistribution as drivers of oxidative stress effects) and biological effects to delineate safer IONPs (215).

Many other stimuli could be proposed to induce M1 polarization in leishmaniasis immunotherapy (217). They should be associated with nanocarriers to favor their selective delivery to macrophages. IONPs emerge as an all-in-one solution as the carriers themselves produce M1 bias to a greater or lesser extent depending on the immune microenvironment.

## Author contributions

CP-C: Conceptualization, Writing – original draft, Writing – review & editing. EM: Writing – review & editing. JI: Writing – review & editing. SE: Writing – review & editing, Conceptualization, Funding acquisition, Supervision, Writing – original draft.
